# The roles of the “ventral” semantic and “dorsal” pathways in *conduite d'approche:* a neuroanatomically-constrained computational modeling investigation

**DOI:** 10.3389/fnhum.2013.00422

**Published:** 2013-08-26

**Authors:** Taiji Ueno, Matthew A. Lambon Ralph

**Affiliations:** ^1^Neuroscience and Aphasia Research Unit, School of Psychological Sciences, University of ManchesterManchester, UK; ^2^Japan Society of the Promotion of ScienceTokyo, Japan

**Keywords:** repetition, dual dorsal-ventral pathway, conduite d'approche, semantics, computational modeling

## Abstract

Ever since the 19th century, the standard model for spoken language processing has assumed two pathways for repetition—a phonological pathway and a semantic pathway—and this idea has gained further support in the last decade. First, recent *in vivo* tractography studies have demonstrated both the “dorsal” (via arcuate fasciculus) and “ventral” (via extreme capsule and uncinate fasciculus) pathways connecting from the primary auditory area to the speech-motor area, the latter of which passes through a brain area associated with semantic processing (anterior temporal lobe). Secondly, neuropsychological evidence for the role of semantics in repetition is *conduite d'approche*, a successive phonological improvement (sometimes non-improvement) in aphasic patients' response by repeating several times in succession. Crucially, conduite d'approche is observed in patients with neurological damage in/around the arcuate fasciculus. Successful conduite d'approche is especially clear for semantically-intact patients and it occurs for real words rather than for non-words. These features have led researchers to hypothesize that the patients' disrupted phonological output is “cleaned-up” by intact lexical-semantic information before the next repetition. We tested this hypothesis using the neuroanatomically-constrained dual dorsal-ventral pathway computational model. The results showed that (a) damage to the dorsal pathway impaired repetition; (b) in the context of recovery, the model learned to compute a correct repetition response following the model's own noisy speech output (i.e., successful conduite d'approche); (c) this behavior was more evident for real words than non-words; and (d) activation from the ventral pathway contributed to the increased rate of successful conduite d'approche for real words. These results suggest that lexical-semantic “clean-up” is key to this self-correcting mechanism, supporting the classic proposal of two pathways for repetition.

## Introduction

Classic 19th century neurologists explained various kinds of language impairment (aphasia) by associating a specific language function to different neural substrates, via accumulated human post mortem data, and construction of neuroanatomical models of language processing (see, Weiller et al., 2011, for direct translation). During this era, Wernicke (Eggert, 1977) and Lichtheim (1885) proposed two pathways from “auditory word images” to “motor word images” for repetition: a phonological pathway and a semantic pathway. According to Wernicke, an inability to repeat despite preserved speech comprehension/production was termed *conduction aphasia* and argued that this reflected a failure in the direct, phonological (non-semantic) pathway. Based on his dual-pathway model, Wernicke predicted a possible patient profile, comprised of an inability to repeat pseudowords accompanied by semantically-related errors in real word repetition, which would be a signature of the impact of semantics in repetition (Eggert, 1977). In recent times, of course, this type of patient has been reported in many studies (Michel and Andreewsky, 1983; McCarthy and Warrington, 1984; Butterworth and Warrington, 1995), and termed *deep dysphasia* by Michel and Andreewsky (1983). Another piece of evidence for the role of semantics in repetition is *conduite d'approche*, a progressive phonological improvement in conduction aphasic patients' responses by repeating several times in succession (Shallice and Warrington, 1977; Joanette et al., 1980; Kohn, 1984; Köhler et al., 1998; Saffran, 2000; Nadeau, 2001; Jacqueline and Marta Sarolta, 2008; Berthier et al., 2012), a behavior that is observed most often in the post-recovery phase (Köhler et al., 1998; Franklin et al., 2002; Jacqueline and Marta Sarolta, 2008). Nadeau (2001), amongst others, suggested that this behavior reflects a “clean-up” self-correcting mechanism underpinned by intact lexical-semantic information. Consistent with this hypothesis, successful conduite d'approche sequences are observed for semantically-intact patients (Köhler et al., 1998; Franklin et al., 2002), but not for semantically-impaired jargon aphasia (Butterworth, 1979; Nadeau, 2001). Moreover, this behavior is more common for real words rather than non-words (Kohn, 1984; Köhler et al., 1998; Nadeau, 2001; Franklin et al., 2002). Nadeau (2001) further elaborated Wernicke's dual pathways model and called for an implemented computational model with as much neuroanatomical constraints as possible (O'Reilly, 1998) in order to understand how both intact and aphasic behaviors emerge from a neuroanatomical brain model. The current study delivers Nadeau's request by simulating conduite d'approche using a model under the constraints from contemporary neuroanatomical data (Ueno et al., 2011).

**Figure 1:**
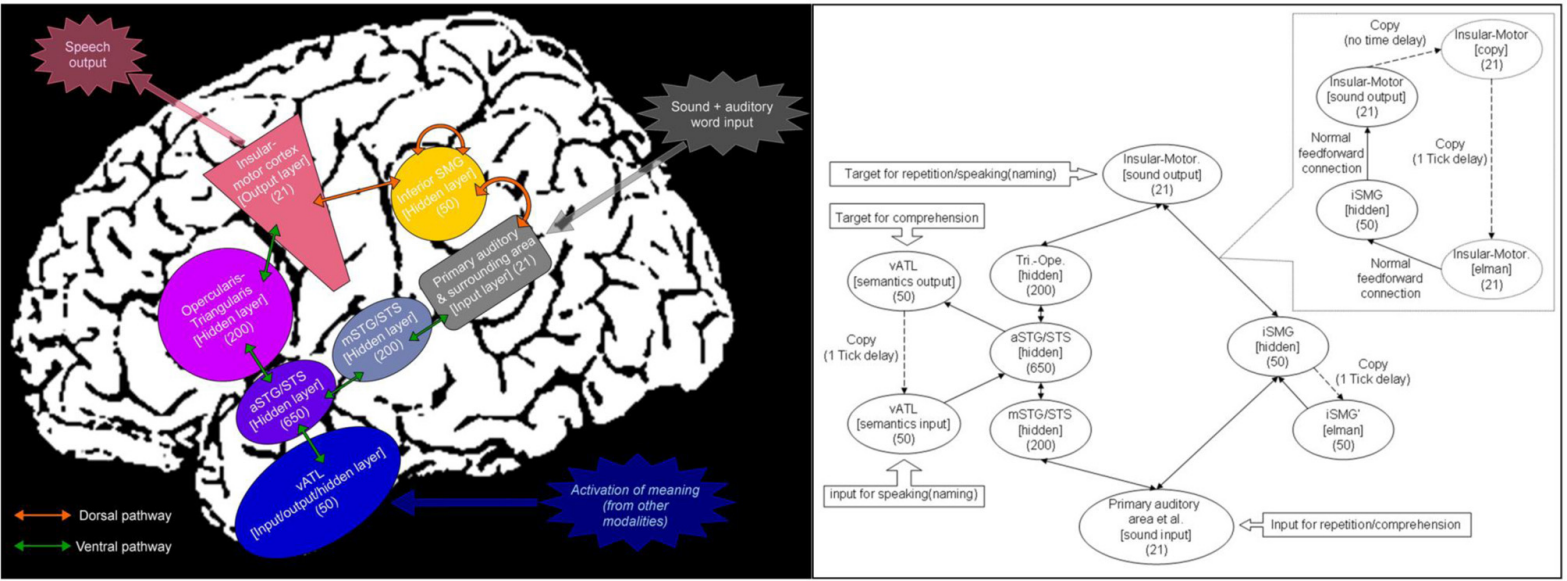
**Implemented neuroanatomically-constrained dual-pathway language model (left), and its exact architecture and activation flow in a simple-recurrent Elman network model (right).** Note. See Ueno et al. (2011) for the full implementation details.

Recent developments in neuroimaging have contributed to more precise neuroanatomical models of language function than those from the 19th century. Historically, the neural correlates of auditory language processing have been dominated by deliberation of the posterior superior temporal lobe (Wernicke's area), the inferior frontal lobe (Broca's area) and their connection via the direct *dorsal* pathway along the arcuate fasciculus, which subserves sound-motor mapping (Lichtheim, 1885; Geschwind, 1970; Eggert, 1977, but see, Weiller et al., 2011 for the direct translation of Wernicke's work). In the contemporary literature, there is an increasing number of studies that also implicate a *ventral* pathway connecting auditory regions, the anterior part of temporal lobe and prefrontal areas via the *extreme capsule complex* and/or the *uncinate fasciculus* (Hickok and Poeppel, 2004, 2007; Parker et al., 2005; Saur et al., 2008; Rauschecker and Scott, 2009; Binney et al., 2012). In contrast to the sound-motor mapping of the dorsal pathway, this ventral pathway has been associated with realization of a sound-meaning-motor mapping (Parker et al., 2005; Hickok and Poeppel, 2007; Saur et al., 2008; Rauschecker and Scott, 2009). These dual dorsal-ventral pathways models have indicated that both pathways work together to support language activities with a functional division of labor. Previously, we constructed a computational model by carefully mirroring this dual pathway neuroanatomy (Ueno et al., 2011) as well as assimilating important knowledge from the past computational models of cognitive/language processing (McClelland et al., 1989; Seidenberg and McClelland, 1989; Plaut et al., 1996; Dell et al., 1997; Joanisse and Seidenberg, 1999; Plaut and Kello, 1999; Harm and Seidenberg, 2004; Rogers et al., 2004; Botvinick and Plaut, 2006; Woollams et al., 2007; Dilkina et al., 2008, 2010; Sibley et al., 2008; Nozari et al., 2010; Welbourne et al., 2011; Yang et al., 2013). The resultant dorsal-ventral dual pathway neurocomputational model (see Figure 1) successfully simulated both normal and impaired language behaviors within a single neurocomputational framework (Ueno et al., 2011). In line with the original Wernicke-Lichtheim ideas, the dorsal pathway of this model developed to be more crucial for phonological processing whereas the ventral pathways was relatively dedicated to semantically-related processing (speech comprehension and speech production from meaning).

If this model is a successful one, then it should be able to simulate additional neuropsychological evidence regarding the claim for two repetition pathways. For this purpose, the current model was tested on its ability to simulate conduite d'approche. Given the division of labor across pathways in the model (Ueno et al., 2011) and the neuropsychological evidence noted above (Butterworth, 1979; Kohn, 1984; Köhler et al., 1998; Franklin et al., 2002; Jacqueline and Marta Sarolta, 2008), the following specific predictions can be made: (a) damage to the dorsal pathway of the model should result in conduction aphasia; (b) following recovery, the model should show more successful conduite d'approche sequences, indexed by the ratio of successfully repeated items in the second trial to the incorrectly repeated items in the first trial; (c) this should be more frequent for real words than for non-words; and finally, (d) successful conduite d'approche should be supported partly by intact lexical-semantic information from the ventral pathway. As such, a diagnostic lesioning analysis to the ventral pathway of the model should reduce the frequency of successful conduite d'approche, particularly for real words.

## Materials and methods

### Model architecture and activation flow

The left half of Figure 1 summarizes the architecture of the neuroanatomically-constrained model, whilst the right panel shows the exact computational architecture of the parallel-distributed processing (PDP) model for Japanese spoken language processing (Ueno et al., 2011). This is a simple-recurrent Elman network model, in which upward arrows represent feedforward connections and downward ones denote the recurrent connections (Elman et al., 1996; Plaut and Kello, 1999; Sibley et al., 2008). We used the Light Efficient Neural Simulator (LENS software) to implement the model (Rohde, 1999).

### Training (development), tasks, and parameters

The simulation was divided into two phases. The first stage was the developmental phase, where the network was trained on three language tasks (see the upper half of Table 1) until its performance matched that of human adults. The implementation in this phase (e.g., items, parameters, materials) was exactly the same as that employed in Ueno et al. (2011), and readers are referred to the previous paper for further details (for both the procedure and the results). The focus of the current study was the post-recovery conduite d'approche data (see next section).

**Table 1:** **Time course of the trained tasks in the development phase (upper half) and in the recovery phase (lower half)**.

**Tasks**	**Input pattern**	**Target pattern**
**(1) DEVELOPMENT PHASE**		
Standard repetition	3-mora sound vector pattern (1st–3rd time event)	3-mora sound vector pattern (4th–6th time event)
Comprehension	3-mora sound vector pattern (1st–3rd time event)	Semantic vector pattern (1st–3rd time event)
Speaking/Naming	Semantic vector pattern (1st–3rd time event)	3-mora sound vector pattern (1st–3rd time event)
**(2) RECOVERY PHASE**		
Standard repetition	3-mora sound vector pattern (1st–3rd time event)	3-mora sound vector pattern (4th–6th time event)
Conduite d'approche	3-mora sound vector pattern (1st–3rd time event)	3-mora sound vector pattern (7th–9th time event)^*^
Comprehension	3-mora sound vector pattern (1st–3rd time event)	Semantic vector pattern (1st–9th time event)
Speaking/Naming	Semantic vector pattern (1st–3rd time event)	3-mora sound vector pattern (1st–3rd time event)

* *Since the network was also trained on standard repetition, the network automatically produced the auditory output during the 4th–6th time events as well. This means the network was trained to repeat twice. If the second repetition was correct whilst the first one was incorrect, this was counted as successful conduite d'approche behavior. See main text for details*.

One detail of the previous simulations is worth rehearsing here (the time course of the trained tasks during development), in order that a new implementation method reported below (for simulating post damage recovery) is easier to follow. As listed in the upper half of Table 1, the temporal dynamics of the trained tasks were divided into three or six discrete time events. First, in repetition, an auditory word input (3-mora^1^ sequence) was presented sequentially, one mora per event (in the 1st–3rd time events). After this, the network was required to reproduce this time-varying auditory sequence in the same order, one mora per event (in the 4th–6th time events). In auditory comprehension, an auditory word input was presented in the same sequential manner (1st–3rd time events), during which the network was pressured to compute the correct semantic pattern as quickly as possible. In other words, the network was trained to compute the target semantic pattern at every time event. Finally, in speaking/naming, a semantic pattern was presented for three time events (1st–3rd time events), during which the network generated the correct 3-mora sequence (the phonological form for that meaning) in the correct order, one mora per time event. The development phase consisted of 200 epochs of training. During every epoch, each word appeared once for repetition, three times for comprehension and two times for speaking/naming, in random order. The final performance after training was exactly the same as reported in Ueno et al. (2011).

### Simulated lesioning, recovery, and conduite D'Approche

The second simulation phase targeted lesioning (damage) and the recovery process. Following the neuropsychological literature on conduction aphasia with conduite d'approche behavior, the simulated lesioning was applied to the dorsal pathway of the model. Given that conduite d'approche behavior can be observed without subcortical (arcuate fasciculus) damage (Berthier et al., 2012), simulated damage was applied not only to the connectivity in the model (analogous to the underlying white matter damage) but also to the unit outputs (reflecting the effects of cortical damage). Thus, 20% of the incoming links from the iSMG (supramarginal gyrus) layer to the speech-motor layer were randomly selected and removed, and Gaussian noise was added to the unit outputs of the iSMG layer (*SD* = 0.2). The resultant model became conduction aphasic, replicating the previous results of Ueno et al. (2011, Figure 3, p. 388).

After lesioning to the dorsal pathway, the recovery phase simulation was commenced. The conduction aphasic model was trained on the three language tasks as before with one addition, in order to simulate conduite d'approche behavior. Specifically, training consisted of the following four tasks (see lower half of Table 1 for the temporal dynamics). One task was (standard) repetition which was trained in the same way as during development (i.e., “listening” in the 1st–3rd time events → “reproducing” in the 4th–6th time events). In addition, the model was sometimes trained to reproduce the “heard” auditory sequence *once again* during the 7th–9th time events. In this conduite d'approche trial, the target output pattern was only applied during the 7th–9th time events. Since the model was trained for standard repetition as well (i.e., where the target was applied during 4th–6th time events), the model actually learned to produce the phonological output twice. The focus of the investigation here was whether or not the model was able to produce the correct output in the second trial after the first repetition had been incorrect (i.e., successful conduite d'approche sequence). We should note here that at least some patients, if permitted, will often continue to produce approximations of the target word, potentially ad infinitum. Under everyday circumstances, there are many potential sources of information to help with error monitoring including kinesthetic/articulatory feedback, auditory feedback, and non-verbal feedback from a person's conversation partner. These important observations go beyond the scope of this simulation which, instead, was focused on the basis for the changes between successive approximations of the target word or non-word.

Next, a slight change was added to comprehension trials as well. Given a conduite d'approche trial extended over nine time “ticks,” the network was also trained to maintain the meaning of the “heard” auditory sequence for the same duration. Thus, in this recovery phase, the auditory input for comprehension was presented from the 1st to 3rd time events, but the target semantic pattern was applied from the 1st to the 9th time point. A key issue for this study was whether or not the network recovered to use this semantic information for successful conduite d'approche as an *emergent property* (i.e., the model was not “forced” by the modeler to use this maintained semantic information neither for the first nor the second repetition). The speaking/naming trial was the same as the development phase.

The duration of this recovery phase was 20 epochs (one-tenth of the development phase). In each epoch, each word appeared once for standard repetition, once for conduite d'approche, three times for comprehension, and two times for speaking. The network performance was evaluated every five epochs (Figure 3). Learning rate was set to 0.1, and weight decay rate was set to 0.0000001. We should note here that all simulation parameters, including learning rate and weight decay are specific to this model—indeed all computational investigations set model-specific values depending on the nature of the training, representations, etc. The crucial thing to note here is that these parameters are fixed for all the simulations generated in this model (both in this paper and its sister publication: Ueno et al., 2011).

### Materials for testing conduite D'Approche, lexicality, and scoring method

**Figure 2:**
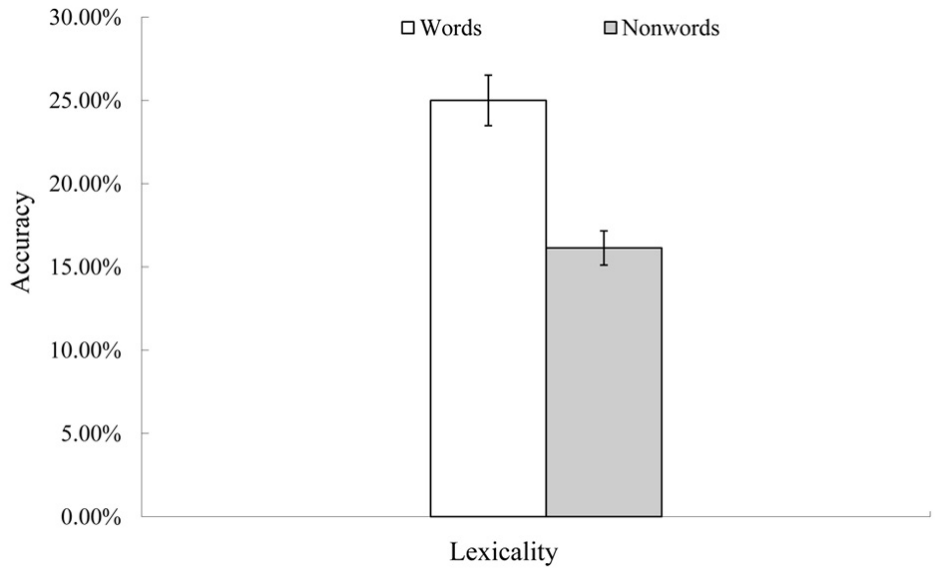
**Rate of successful conduite d'approche post recovery.** Note. Accuracy is expressed as the number of successfully repeated items at the second time (7th–9th time event, see Table 1) divided by the number of incorrectly repeated items at the first time (4th–6th time events). Thus, this is a rate of successful self-correction in the conduite d'approche attempts. *Y*-axis error bars indicate standard errors.

**Figure 3:**
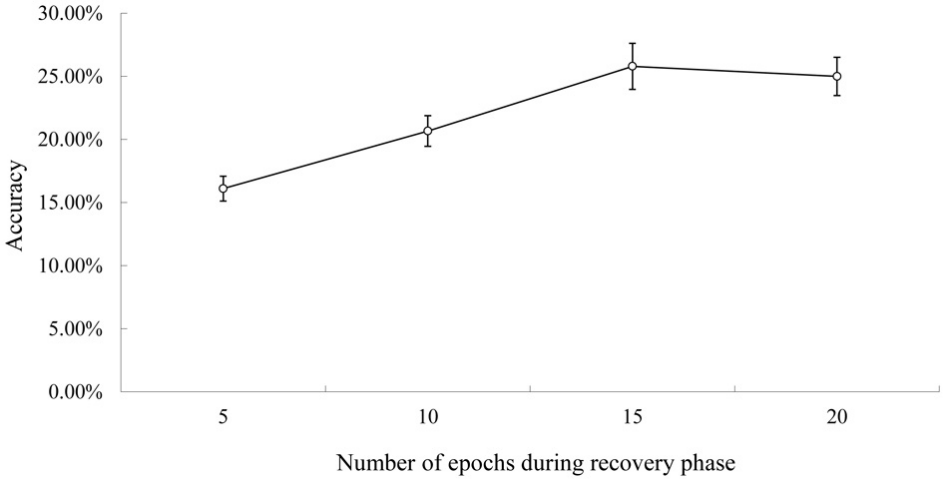
**Rate of successful conduite d'approche during recovery.** Note. Figure 2 for the explanation of accuracy.

**Figure 4:**
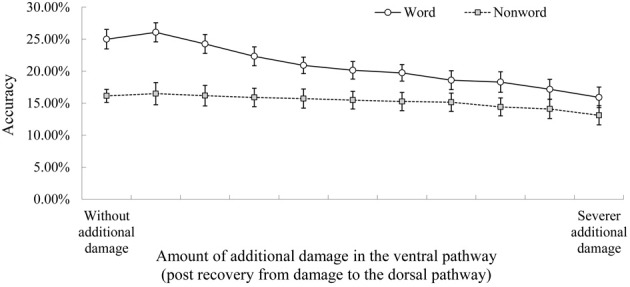
**The effect of subsequent diagnostic damage to the ventral pathway.** Note. See Figure 2 for the explanation of accuracy. The data in the left edge show the performance immediately after recovery from damage to the dorsal pathway (i.e., without the additional diagnostic damage to the ventral pathway).

Throughout the Results section below, we focus on conduite d'approche accuracy post recovery (see, Ueno et al., Figures 2–4, for the performance before damage). A 51-word set and 108-non-word set were created (matched for bimora frequency and Japanese pitch accent types, Tanida et al., 2010) for probing the network's ability to repeat twice. These item sets were used in Ueno et al. (2011, Figure 4, p. 389) to test the lexicality effect. The reported accuracy for conduite d'approche in the subsequent Figures refers to the number of successfully repeated items in the second trial (7th–9th time events, see Table 1) as a proportion of the number of incorrectly repeated items in the first trial (4th–6th time events). Note here that the network was trained to repeat during the 4th–6th time events and during the 7th–9th time events in equal frequency. Therefore, there was not an implementation-specific reason to expect a correct repetition following an incorrect repetition, but rather this successful conduite d'approche behavior was an emergent property.

### Role of semantics (diagnostic lesioning to the ventral pathway)

The role of semantics in conduite d'approche was probed by additional lesioning to the ventral pathway (Figure 4). The same approach was used in Ueno et al. (2011, Figure 4, p. 389). The study-specific parameters were as follows. The post-recovery model was tested for repeating twice as a function of increasing severity of diagnostic damage to the two layers in the ventral pathway (the ventral anterior temporal lobe layer and the anterior superior temporal gyrus/sulcus layer, see Figure 1). Twenty levels of damage severity were simulated by adding an increasing amount of noise to the output of these two layers (ranging from 0.01 to 0.2 to vATL layer and from 0.005 to 0.1 to aSTG/STS layer, in equal intervals) and by removing an increasing proportion of the links incoming to these layers (from 0.5 to 10% to vATL layer and from 0.25 to 5% to aSTG/STS layer).

### Statistical analysis

The procedure described above was conducted on 10 randomly initialized (different initial weights) networks (as an analogy of collecting 10 participants in human experiments), and data from these 10 models were entered into a statistical analysis. Each damaged model was probed with random noise five times and, as a result, performance of the damaged model was measured 50 times and averaged for a stable outcome.

## Results

### Conduite D'Approche post recovery

Figure 2 shows the conduite d'approche accuracy after 20 epochs of recovery. Like real patients, successful conduite d'approche was observed mainly for words, rather than non-words, *F*_(1, 9)_ = 34.39, *p* < 0.01. The rate of successful conduite d'approche for words (25%) was close to that of real patients (approximately 30% accurate, Köhler et al., 1998).

### The effect of recovery on successful conduite D'Approche

Figure 3 shows the conduite d'approche accuracy during recovery. The accuracy increased steadily during recovery and reached asymptote around 15th–20th epochs. There was a significant difference in accuracy between the early recovery phase (5th epoch) and the later phase (20th epoch), *F*_(1, 9)_ = 56.87, *p* < 0.01. Thus, like real patients, there was a greater number of successful conduite d'approche attempts post recovery (Köhler et al., 1998; Franklin et al., 2002; Jacqueline and Marta Sarolta, 2008).

### The role of the “ventral” semantic pathway in conduite D'Approche

Figure 4 shows the effect of simulated diagnostic lesioning to the ventral pathway to probe its contribution to the conduite d'approche accuracy. The rationale behind this approach is that if activation from the ventral pathway is crucial, then accuracy should decrease as a function of increasing additional damage to the ventral pathway. This analysis found that the conduite d'approche accuracy for real words was particularly sensitive to damage to the ventral pathway. There was a significant difference in accuracy between the post-recovery model without additional ventral damage (left edge of Figure 4) and that with severest additional damage (right edge of Figure 4) for real words, *F*_(1, 9)_ = 16.03, *p* < 0.01, Cohen's *d* = 2.1. In contrast, the effect of this diagnostic lesioning for non-words was significant, but the effect size was smaller *F*_(1, 9)_ = 9.59, *p* = 0.012, Cohen's *d* = 1.2. The interaction between lexicality and with/without additional ventral damage was significant *F*_(1, 9)_ = 11.09, *p* < 0.01. Therefore, the contribution from the ventral semantic pathway was more crucial for real words than for non-words.

## Discussion

Since the work of Wernicke and Lichtheim, the neuroanatomical model for spoken language processing has proposed two pathways for repetition—a phonological pathway and a semantic pathway (Lichtheim, 1885; Eggert, 1977). More recently, this notion has been supported both by contemporary neuroanatomical data (tractography) that have demonstrated a ventral white matter pathway that passes through semantically-related brain areas (Hickok and Poeppel, 2004, 2007; Parker et al., 2005; Saur et al., 2008; Rauschecker and Scott, 2009; Binney et al., 2012) and by neuropsychological data regarding conduite d'approche in conduction aphasic patients (Shallice and Warrington, 1977; Joanette et al., 1980; Kohn, 1984; Köhler et al., 1998; Saffran, 2000; Nadeau, 2001; Franklin et al., 2002; Jacqueline and Marta Sarolta, 2008; Berthier et al., 2012) Nadeau (2001), amongst others, hypothesized that this conduite d'approche behavior is underpinned by a lexical-semantic mechanism which “cleans up” noisy phonology activation, and called for computational modeling to demonstrate this idea. The current study aimed to test this hypothesis by using a neuroanatomically-constrained dual dorsal-ventral pathways computational model (Ueno et al., 2011).

The current simulation closely mirrored data from conduction aphasic patients with a conduite d'approche behavior. First, damage to the dorsal pathway of the trained (adult) model gave rise to conduction aphasia (Ueno et al., 2011). The model was then re-exposed to the learning environment in order to simulate post recovery data (Welbourne and Lambon Ralph, 2007). Following this simulated partial recovery, the model acquired an ability to correct its own first repetition (successful conduite d'approche). As with real patients, successful conduite d'approche was mainly observed for real words than for non-words, and accuracy was equivalent to real patients (approximately 30% of successful conduite d'approche within the attempts made). Finally, diagnostic lesion analysis demonstrated that activation from the ventral semantic pathway contributed to successful conduite d'approche particularly for real words.

As noted above, previous researchers have accumulated detailed data regarding conduite d'approche and discussed this self-correcting mechanism in terms of a proposed lexical-semantic “clean-up” (Kohn, 1984; Köhler et al., 1998; Nadeau, 2001; Franklin et al., 2002). Our computationally-implemented demonstration of the impact of ventral semantic pathway to conduite d'approche has advanced this theory more explicitly. In repetition, once an auditory input is heard, the corresponding semantic pattern is activated in the ventral semantic layer (i.e., is automatically comprehended). This activation in the semantic system then gradually propagates to the other layers in the ventral pathway, and eventually to the dorsal pathway as well. Given that repetition primarily relies on the phonological system rather than the semantic system (as is clear from our ability to repeat a pseudoword), activation from the dorsal, phonological pathway has a main (yet not modular) role in immediate repetition for neurologically-intact people and model (Fridriksson et al., 2010; Buchsbaum et al., 2011; Ueno et al., 2011). However, as reproduction of the auditory input is attempted twice (or more) in conduite d'approche, activation from the ventral pathway has a chance to interact further with the dorsal pathway and, as a result, the errorful repetition output from the first trial can be moved toward the correct target.

Our simulation can also explain why successful conduite d'approche in conduction aphasic patients is more readily observed in the post-recovery phase (Köhler et al., 1998; Franklin et al., 2002; Jacqueline and Marta Sarolta, 2008). The recent neuroimaging/neuropsychological/modeling literature has demonstrated that an impaired function can be at least partially re-acquired by reorganizing the function of the remaining brain areas (Leff et al., 2002; Saur et al., 2006; Welbourne and Lambon Ralph, 2007; Sharp et al., 2010). In terms of the dual dorsal-ventral pathway framework, Ueno et al. (2011) found that this recovery process could be supported, at least in part, by a changed interaction between the two pathways (see also Welbourne et al., 2011). Specifically, the intact model acquired a division of labor between the two pathways as an emergent property, such that the dorsal pathway was more crucial for phonological processing and the ventral pathway for semantic processing. Consequently, damage to the dorsal pathway impairs activities that heavily tap phonological processing such as repetition, resulting in conduction aphasia. Then, as the model is allowed to reorganize the remaining intact resources in the ventral pathway, some computational aspects of repetition can be shifted to the ventral pathway (Ueno et al., 2011). This plasticity-related post-recovery shift in division of labor (Welbourne and Lambon Ralph, 2007) might be a crucial foundation for lexical-semantic information to serve conduite d'approche.

Although not focused on rehabilitation per se, the current simulations provide some ideas for potential intervention strategies that could be formally assessed. Specifically, the model improves its repetition performance by (a) increasing the role of the semantically-imbued ventral pathway; (b) involving word meaning more in spoken production; which, (c) increases over time (allowing semantically-driven activation to percolate through the remaining language system). For behavioral interventions, this would suggest that, when repeating, it might be beneficial (i) to encourage processing of the target's meaning as well as phonological form in this type of aphasic patient and (ii) also to discourage instantaneous, echolalic-like responding which may not allow sufficient time for word meaning to influence the spoken production. In addition, if transcranial stimulation techniques prove to be an effective method to modulate the functioning of the remaining language neural network, then it might be possible to enhance the relative contribution of the ventral (semantic) pathway, if still intact, and thus, improve the performance on real word production. For both rehabilitation approaches, we should note that the consequence of increased semantic contribution to repetition and phonological processes more generally, is that it will improve accuracy on real words but that the ability to generalize the acoustic-motor speech statistics and thus deal with non-word items will diminish, leading to increasing lexicalization errors (see Ueno et al., 2011 for a formal demonstration of this effect during simulated recovery).

Whilst the current simulation successfully demonstrated conduite d'approche behavior within the dual dorsal-ventral pathways neurocomputational framework, there are other relevant phenomena to be explained in future studies. For example, the current simulation does not clarify why conduite d'approche attempts sometimes deviate away from the target pattern in patients and why patients do not always make such attempts (Köhler et al., 1998; Saffran, 2000; Franklin et al., 2002). In addition, conduction aphasic patients show conduite d'approche in spontaneous speaking as well, not just in repetition (Franklin et al., 2002). Explaining these phenomena will be future targets.

### Conflict of interest statement

The authors declare that the research was conducted in the absence of any commercial or financial relationships that could be construed as a potential conflict of interest.

## Footnotes

^1^The mora is a subsyllabic spoken unit in Japanese. Morae include all of the following types of elements: a vocalic nucleus (V), a nucleus with onset (CV or CCV), a nasal consonant (N) in syllabic coda position, a geminate consonant or a long vowel.
